# Enhancing the get healthy information and coaching service for Aboriginal adults: evaluation of the process and impact of the program

**DOI:** 10.1186/s12939-017-0641-8

**Published:** 2017-09-06

**Authors:** E. Quinn, B. J. O’Hara, N. Ahmed, S. Winch, B. McGill, D. Banovic, M. Maxwell, C. Rissel

**Affiliations:** 1 Public Health Unit, Sydney Local Health District, Level 9, King George Building, RPAH, Missenden Road, Camperdown, 2050 Australia; 2 Prevention Research Collaboration, Sydney School of Public Health, University of Sydney, Level 6, The Hub, Charles Perkins Centre D17, 2006 Camperdown, Australia; 3 NSW Office of Preventive Health, NSW Ministry of Health, Liverpool Hospital, Level 1, Don Everett Building, West End, Elizabeth Street, Liverpool, NSW 2170 Australia; 4 School of Medicine, University of Wollongong, Northfields Avenue, Wollongong, NSW 2540 Australia; 5 Planning Unit, Sydney Local Health District, KGV Missenden Road, Camperdown, NSW 2050 Australia; 6 Centre for Population Health, NSW Ministry of Health, 73 Miller Street, North Sydney, 2060 Australia; 7 NSW Office of Preventive Health, NSW Ministry of Health and Prevention Research Collaboration, Sydney School of Public Health, University of Sydney, Camperdown, Australia; 8 NSW Office of Preventive Health, Liverpool Hospital, Level 1, Don Everett Building, West End, Elizabeth Street, Liverpool, NSW 2170 Australia

**Keywords:** Aboriginal communities, Consultation, Community engagement, Telephone-counselling, Lifestyle risk factors

## Abstract

**Background:**

Non-communicable chronic diseases in Australia contribute to approximately 85% of the total burden of disease; this proportion is greater for Aboriginal communities. The Get Healthy Service (GHS) is effective at reducing lifestyle-based chronic disease risk factors among adults and was enhanced to facilitate accessibility and ensure Aboriginal cultural appropriateness. The purpose of this study is to detail how formative research with Aboriginal communities was applied to guide the development and refinement of the GHS and referral pathways; and to assess the reach and impact of the GHS (and the Aboriginal specific program) on the lifestyle risk factors of Aboriginal participants.

**Methods:**

Formative research included interviews with Aboriginal participants, leaders and community members, healthcare professionals and service providers to examine acceptability of the GHS; and contributed to the redesign of the GHS Aboriginal program. A quantitative analysis employing a pre-post evaluation design examined anthropometric measures, physical activity and fruit and vegetable consumption of Aboriginal participants using descriptive and chi square analyses, t-tests and Wilcoxon signed-rank tests.

**Results:**

Whilst feedback from the formative research was positive, Aboriginal people identified areas for service enhancement, including improving program content, delivery and service promotion as well as ensuring culturally appropriate referral pathways. Once these changes were implemented, the proportion of Aboriginal participants increased significantly (3.2 to 6.4%). There were significant improvements across a number of risk factors assessed after six months (average weight loss: 3.3 kg and waist circumference reduction: 6.2 cm) for Aboriginal participants completing the program.

**Conclusions:**

Working in partnership with Aboriginal people, Elders, communities and peak bodies to enhance the GHS for Aboriginal people resulted in an enhanced culturally acceptable and tailored program which significantly reduced chronic disease risk factors for Aboriginal participants. Mainstream telephone based services can be modified and enhanced to meet the needs of Aboriginal communities through a process of consultation, community engagement, partnership and governance.

## Background

Approximately 85% of the total burden of disease in Australia is associated with chronic diseases [1]. Further, 70% of the health disparity between Aboriginal and non-Aboriginal people can also be attributed to chronic diseases [2] and heart disease and diabetes are the top two contributors to this gap [3]. For example, Aboriginal adults are 1.6 times more likely to experience cardiovascular disease, and 1.7 times more likely to experience stroke than non-Aboriginal adults and 2.7 times more likely to experience diabetes [4].

The chronic diseases mentioned above share many of the same risk factors e.g. high fat, sugar and low nutritional intake diets, low levels of physical activity and overweight and obesity [3]. Therefore these chronic diseases can be prevented by modifying these behavioural or lifestyle risk factors [1, 5, 6]. This is particularly relevant for Aboriginal communities, where evidence indicates that Aboriginal peoples are not meeting the recommended daily guidelines for healthy eating and physical activity [3, 7, 8]. For example, almost two thirds of Aboriginal people reported no or low levels of physical activity per day, consume less fruit and experience higher obesity rates (1.5 times rate for non-Aboriginal people) [3].

However, it is also important to acknowledge that the complex interplay between personal, social and environmental resources can disadvantage some Aboriginal peoples and negatively impact their health [9]. For example, low household incomes may make it difficult for some Aboriginal families to purchase nutritious food, or participate in sport or exercise programs through lack of access to public or private transport [10].

Telephone-based interventions have demonstrated effectiveness in increasing physical activity, improving nutrition and reducing weight in the short to medium term (three-six months) across different populations, in a range of settings, and using different intervention modalities [11, 12]. Research also suggests that such interventions targeting lifestyle risk factors in Aboriginal communities could contribute to positive health outcomes for Aboriginal Australians [13].

The New South Wales (NSW) Get Healthy Service (GHS) is a free telephone-based service supporting NSW adults to make sustained improvements in healthy eating, physical activity, reducing alcohol intake and achieving or maintaining a healthy weight. The GHS targets adults in the community most at need, due to their risk of chronic disease, and seeks population level reach to maximise its public health impact [14]. The service provides information, where participants are sent detailed information on healthy eating, active living and achieving or maintaining a healthy weight; and also the opportunity to participate in 10 coaching calls over six months, where participants are supported by a coach to set and meet lifestyle related goals and change lifestyle behaviours. The NSW GHS has demonstrated its effectiveness at supporting individuals to positively change their behaviours and reduce their risk factors for chronic disease [15]. The service has also demonstrated its reach for population groups most in need of support, including people living in rural and remote communities, people from low socioeconomic backgrounds and Aboriginal people in NSW [16].

Central to the development of any program and service targeting Aboriginal community members are principles of community engagement, self-determination at all phases of development to help ensure feasible, effective and culturally appropriate interventions which are built in partnership with Aboriginal communities and their healthcare services [17–20]. With these best practice principles in mind, an Aboriginal-specific enhancement to the GHS was planned, implemented and evaluated. It should be noted that Australia has historically been inhabited by two ethnically and culturally different indigenous peoples, Aboriginal and Torres Strait Islander peoples, who both have diverse nations, languages and traditions [21, 22]. However, in NSW, Australia, the term Aboriginal peoples is generally recognised as the term to represent both Aboriginal and Torres Strait Islander peoples, in recognition that Aboriginal people are the original inhabitants of NSW [21, 22].

The aims of this paper are to (a) describe how the formative research [the exploratory study and the appropriateness study] with Aboriginal communities was applied to guide the development and refinement of the GHS; and (b) assess the reach and impact of the GHS (and the Aboriginal specific program) on the lifestyle risk factors of Aboriginal participants.

## Methods

### Governance and development of the GHS Aboriginal program

**Fig. 1:**
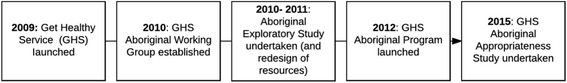
Aboriginal GHS program development timeline

The development, delivery and effectiveness of the mainstream GHS have previously been reported [14]. The development and implementation of the GHS Aboriginal program was informed by formative exploratory research with Aboriginal people, communities and peak bodies. This formative work was governed by an Aboriginal Working Group, comprised of the NSW Aboriginal Health and Medical Research Council (AHMRC, the peak body representing Aboriginal peoples and medical and health services in NSW), General Practice NSW (at the time, the peak body representing general practitioners across NSW), the GHS provider at the time, i.e. Medibank Health Solutions and, NSW Ministry of Health (MoH) personnel, with other stakeholders providing advice as needed (Fig. 1).

#### Qualitative formative research – exploratory study

The NSW MoH commissioned a consultancy firm i.e. Cultural Partners Australia (in conjunction with Origin Communications) to undertake an explorative qualitative study to understand how the mainstream GHS could better meet the health and cultural needs of Aboriginal people [23]. The methods used in this exploratory study were based on relevant Australian Social and Market Research Association [24] and Australian Institute of Aboriginal and Torres Strait Islander Studies Guidelines for Ethical Research in Indigenous Studies [25] and represented best practice standards in Aboriginal research design and implementation. This formative research focused on three objectives: (i) the GHS concept and fit for Aboriginal people; (ii) Aboriginal specific marketing and communication strategies; and the (iii) experiences of Aboriginal community members who had used the GHS. The study included current and previous GHS participants taking part in telephone and face-to-face (ratio 5:1) interviews. Community consultations were conducted with Aboriginal community members who would be the target audience for the GHS through mini group discussions and face-to-face in-depth interviews were also conducted with Aboriginal community leaders, healthcare professionals and associated service providers.

Aboriginal GHS participants who had consented to being contacted for the purposes of evaluation were recruited through a sample list provided by the NSW MoH (*n* = 64: previous or current participants) and contacted by phone to seek their voluntary involvement in the study and consent was obtained. Interviews took approximately 30–40 minutes using a prepared questionnaire and discussion guide. Focus group discussions were held with community members with and without chronic disease risk factors; and included a socio-demographically diverse range of participants. The interviews and focus group discussions were analysed thematically to identify the acceptability and areas of success or improvement for the current mainstream model and its adaptation for Aboriginal people.

#### Qualitative formative research – appropriateness study

An appropriateness study, commissioned by the NSW MoH, was undertaken by the Cultural and Indigenous Research Centre Australia (CIRCA) [26] to assess the appropriateness of the GHS for Aboriginal participants and to identify further opportunities to improve and enhance the GHS. Ethics approval to conduct the study was obtained from the Aboriginal Health and Medical Research Council Ethics Committee. Potential participants (*n* = 101) were sent a letter and an information sheet and then contacted by phone to seek their involvement in the study, highlighting voluntary participation and to obtain consent. Interviews were conducted by CIRCA research consultants (including Aboriginal research consultants) using a prepared questionnaire and discussion guide, and took approximately 30–45 minutes. Thematic analysis was also undertaken to identify the key themes related to the appropriateness of the new changes proposed to enhance the mainstream GHS for Aboriginal people.

#### Quantitative study

The quantitative study employed a pre- and post-test evaluation design, which included collection of data on all service participants at enrolment and then at completion of their three or six month coaching program. Data was collected on all service participants between February 2009 and December 2015 who had provided consent for their data to be included in the evaluation. Ethics approval for this study was granted by the University of Sydney Human Research Ethics Committee (Ref. No. 02-2009/11570).

### Socio-demographic variables

All measures were collected using computer-assisted telephone interviews (CATI) by GHS coaches as previously described here [14]. Information on gender, date of birth, residential postcode, education level, employment status, language spoken at home and Aboriginal status were collected using questions from the NSW Population Health Survey [27]. Participants’ postcodes were used to determine Socio-Economic Indexes for Areas (SEIFA) [28], as a measure of area socio-economic status, and Accessibility-Remoteness Index of Australia Plus (ARIA) as a measure of geographical location remoteness [29]. The SEIFA data are based on aggregate area-level socio-economic status indicators and were categorized into quintiles (1 = most advantaged, 5 = most disadvantaged). The ARIA is categorized as major cities, inner regional, outer regional, remote and very remote.

### Outcome measures

The primary anthropometric measures were self-reported weight (kg), height (cm), and waist circumference (cm), which were asked using a standard script. Height and weight were used to calculate BMI and then classified into: underweight (<18.49), acceptable weight (18.5–24.99), overweight (25.00–29.99) and obese (≥30.00) [30]. Waist circumferences risk categories were calculated for males (<94 cm no risk; increased risk ≥94 cm to <102 cm; greatly increased risk ≥102 cm); and for females (<80 cm no risk; increased risk ≥80 cm to <88 cm; and greatly increased risk ≥88 cm) [31]. Physical activity was assessed by three validated questions, which asked about number of weekly walking sessions, moderate-intensity physical activity for 30 minutes or more; and vigorous-intensity physical activity for 20 minutes or more [32, 33]. Categories for recommended physical activity were defined by those engaging in ≥5 sessions per week of walking, or ≥5 sessions per week of moderate activity, or combinations of walking and moderate-vigorous activity summing to 5 sessions per week [32]. For fruit and vegetable consumption, participants reported consumption of the number of daily serves of fruit and vegetables [34] to GHS coaches. Participants were categorised into those meeting the recommended levels of consumption of ≥2 serves of fruit daily, and ≥5 serves of vegetables daily in accordance with Australian Dietary Guidelines [34].

### Data management and statistical analysis

Descriptive and chi square analyses were performed [IBM SPSS Statistics 22 Inc. 2009] on key socio- demographic variables stratified by the type of GHS program the participants enrolled in; time period [pre and post November 2013, the time of the introduction of the Aboriginal program]; and their Aboriginal status. Matched [within-individual] paired t-tests were performed to examine changes in weight, waist, and BMI from baseline to follow-ups, as these data followed normal distributions. Wilcoxon signed-rank tests were performed to examine changes in fruit and vegetable intake as these data were non-normally distributed. T-tests comparing differences between groups were also undertaken in relation to changes between baseline and 6-months based on Aboriginal status, program of enrolment and time period.

## Results

### Qualitative formative research – exploratory study

#### Study participants: demographics

Thirty Aboriginal GHS participants were interviewed, with the majority of participants being female (*n* = 25; 84.0%) and 75% (*n* = 23) lived outside greater Sydney. Participants were aged 19–63 years, with 40% (*n* = 12) between 40–54 years. Half (*n* = 15) of the participants had contact with GHS 12 months prior to the interview and 20% (*n* = 6) had contact with GHS in the same year as the interview. Two-thirds (*n* = 20) of the participants had registered for information-only and had not progressed to the coaching program; 33.0% (*n* = 10) of participants had registered for the coaching program, *n* = 1 (3%) did not start the program as they were unable to obtain medical clearance, *n* = 3 (9%) completed the program and *n* = 5 (17%) had completed approximately 3 months of the program; and *n* = 1 (3%) was still enrolled in the program.

Twenty Aboriginal community members who would be considered part of our target GHS population were included in mini group discussions and a further twenty Aboriginal community leaders, healthcare professionals and associated service providers took part in face-to-face in-depth interviews.

#### Key findings

The exploratory research suggested that Aboriginal people would find the service beneficial but not always desirable; and that addressing lifestyle behaviours must consider that food choices and losing weight do not necessarily have a high priority within Aboriginal communities. The formative research suggested that promoting the GHS should use straightforward language and strong Aboriginal visuals and colours; emphasise that it is a free confidential service, working closely with Aboriginal Community Controlled Health Services (ACCHS) and be sensitive to Aboriginal needs by providing a personalised service.

The results also suggested that the lack of referral pathways through the ACCHS may reduce participation by Aboriginal people. Previous GHS Aboriginal participants had indicated the value of increased involvement with ACCHS, potentially encouraging GHS use and ensuring greater recognition and acceptance. Aboriginal community members who had participated in the GHS coaching program felt that coaches were helpful, understanding and offered regular support and would recommend the GHS to others. Those that had not progressed to the coaching program expressed concerns about the time commitment required, issues regarding flexibility of booking coaching calls, the ability of the GHS to sensitively understand Aboriginal people, kinship and culture and be responsive to Aboriginal health needs.

#### Service redesign and delivery

Following the exploratory formative research, the working group agreed to a number of GHS enhancements (Table 1). These were implemented with the service provider at the time (Medibank Health Solutions) in November 2012 and launched by the NSW Ministry for Health in January 2013.

**Table 1:** Summary of changes to enhance the GHS for Aboriginal communities

Focus area	Mainstream GHS	Enhanced Aboriginal program
Service design and delivery		
Aboriginal-specific ‘program’ of the GHS is designed for people identifying as Aboriginal and/or Torres Strait Islander	All people are asked whether they identify as being Aboriginal and/or Torres Strait Islander. People identifying as Aboriginal follow the same call flow and coaching process as non-Aboriginal people.	Aboriginal participants are identified according to best practice guidelines in NSW and they can register for Level 1 [information-only] or Level 2 [health coaching] support, but now receive Aboriginal-specific information materials and three additional coaching calls
Aboriginal-specific program resources	People who register to receive information-only or to participate in the coaching program are sent the following resources: • Welcome letter from the Chief Health Officer • GHS information booklet and/or coaching journal	When an Aboriginal participant is identified, Aboriginal-specific resources are sent to these participants. • Welcome letter from the Chief Health Officer • Aboriginal information booklet and/or coaching journal
Increase in the number of coaching calls from the service	Participants registering with the service receive a total of 10 calls.	Aboriginal participants now receive an additional 3 calls [total 13 calls]. The additional calls are educational sessions with content focused on prevention of diabetes where appropriate.
Increased call attempts from GHS service to participants for coaching sessions	The GHS makes 3 call attempts to contact a participant, if the call attempts are not successful the participant is withdrawn from service [with the distribution of a letter to the participant offering re-enrollment].	For Aboriginal participants 5 call attempts are made.
Training for health coaches	Coaches are trained and their professional development is monitored by the GHS provider.	All health coaches receive annual cultural competency training in addition to the routine education and training provided service provider.
Development of Aboriginal specific referral database	A database is available to all health coaches so that they can refer participants to appropriate health services or organizations in the community.	This database was redeveloped for health coaches, so that they can refer Aboriginal participants to local Aboriginal specific health services and/or organisations across NSW for issues outside the scope of the GHS.
Promotion and referral		
Education of key stakeholders	Mainstream health services and providers were educated regarding the benefits of the GHS.	A series of promotional campaigns, workshops and conferences across NSW were implemented to promote activity through routine chronic disease networks and Aboriginal specific cultural events.
Referral pathways from Aboriginal Community Controlled Health Services [ACCHS] to GHS	People can be referred to the service through the following pathways: • Self-referral; • General practice; and • Other health professional.	In addition to the standard GHS referral pathways, Aboriginal people can be referred by health professionals i.e. Aboriginal Health Workers [AHWs] or others working in the Aboriginal Community Controlled Health Services [ACCHS].
Aboriginal specific promotional material	Promotional materials are not specific to the Aboriginal community	Resources have been developed specifically for the Aboriginal community, including print, online and radio advertisements and a number of resources targeting pregnant Aboriginal women and these are available through the GHS website (http://www.gethealthynsw.com.au/professionals-resources)

While the service enhancements mainly refer to telephone service changes, three key enhancements were made to ensure cultural appropriateness. These included a referral pathway through the ACCHS with Aboriginal Health Workers seen as key educators in chronic disease prevention and facilitators in referring clients to the service. All GHS coaches would attend annual cultural competency training, to ensure a culturally sensitive and respectful service [35]. Furthermore, given the diversity of Aboriginal peoples in NSW and the unique kinship and family relationships between different nations, the feedback from the exploratory study also indicated the need for GHS coaches to have access to an online database enabling them to refer participants to local Aboriginal-specific health services and programs across NSW for issues outside the scope of the GHS.

#### Service promotion

In order to build awareness and credibility of the enhanced GHS for Aboriginal peoples, there was a clear need to first educate key stakeholders about the benefits of the service (Table 1).

This involved presentations at workshops and conferences across NSW prior to the launch of the service e.g. Closing the Gap workshop with General Practice NSW and the AHMRC Chronic Disease Conference in 2012.

The working group also endorsed the principle of establishing partnerships with the AHMRC to support the ACCHS to promote the service locally through their own established services, programs and networks [36]. After the service was launched in January 2013, the AHMRC received funding to facilitate promotion of the enhanced GHS for Aboriginal peoples, including promotional activity at Aboriginal-specific cultural events e.g. NSW Aboriginal Rugby League Knockout carnival (an Aboriginal rugby tournament held every few years in NSW) and National Aborigines and Islander Day Observance Committee week (Table 1). Cultural Partners^TM^ were also contracted to redesign the existing GHS promotional resources for Aboriginal peoples to ensure they were culturally appropriate under NSW Health guidelines [21] (http://www.gethealthynsw.com.au/professionals-resources ). This included print, online and radio advertisements (Table 1).

### Qualitative formative research – appropriateness study

#### Study participants: demographics

The study included 32 in-depth telephone interviews with Aboriginal GHS participants who had completed, those currently enrolled and those who had withdrawn from the coaching program. The majority were female (*n* = 26; 81.3%) and were aged 30–59 years (*n* = 25; 78.1%). Fourteen participant interviews were conducted in major cities, *n* = 16 in inner and outer regional locations and *n* = 2 in remote locations.

#### Key findings

Feedback about the GHS was overwhelmingly positive [37], with the coaching program highly valued and high satisfaction reported. Participants felt that the program allowed them to set individual goals and provided information and advice related to their individual weight loss challenges [37]. They appreciated the program flexibility and that coaches would follow up and reschedule calls if necessary [37]. Coaches were noted as integral to the success of the program [37].

### Quantitative study

For the period February 2009 to December 2015, 34,211 participants registered their interest in the GHS and consented for their information to be included in the evaluation. For participants with multiple GHS enrolments, this evaluation focused on their first enrolment. Of these participants 99.1% (33,897) were classified according to the GHS programs available, with 28,801 (85.0%) enrolling in the standard GHS service; 4,280 (12.6%) in the Diabetes prevention program; and 816 (2.4%) in the Aboriginal program.

#### Socio-demographic profile of GHS participants

The average age of GHS participants for the mainstream program was 49 years (SD 15.3); the majority were female (74.2%) and 95.5% were non-Aboriginal. There were significant differences between those enrolled in the Aboriginal program only, compared to those enrolled in both the standard GHS program. Participants registered in the GHS Aboriginal program were significantly more likely than participants enrolled in the other programs to be aged 18–49 years (69.4% compared to 49.5%; *p*-value <0.0001); have a high school education (59.2% compared to 43.2%; *p*-value <0.0001); be from a location classified as being most disadvantaged (in the 3rd, 4th or 5th quintile) (85.9% compared to 66.1%; *p*-value <0.0001); and be from inner and outer regional, and remote and very remote locations (63.1% compared to 40.2%; *p*-value <0.0001).

#### GHS participation by Aboriginal community members

For the total period February 2009-December 2015, 4.5% (*n* = 1,462) of all GHS participants (regardless of program type) were Aboriginal. Overall the number of Aboriginal participants increased since 2009; from 2.3% (*n* = 66) in 2009 to 8.8% (*n* = 345) in 2015. Participation by Aboriginal community members significantly increased following the introduction of the GHS Aboriginal program and associated Aboriginal recruitment strategy in November 2012 (*n* = 606, 3.2% compared to *n* = 856, 6.4%: *p*-value < 0.0001).

#### Referral sources by Aboriginal community members

The main referral source for all GHS participants was mass media [59.9%, *n* = 19,104]. Aboriginal participants were significantly more likely to cite health professionals and Aboriginal community health professionals as their source of referral compared to non-Aboriginal participants. Health professionals have increasingly played an important role in promoting the GHS to Aboriginal participants since the inception of the service in 2009, with increases in the proportion of Aboriginal community members being referred to the GHS from 13.6% (*n* = 9) to 76.7% (*n* = 263) in 2015. In relation to referral sources for Aboriginal participants prior to and after the implementation of the GHS Aboriginal program (in November 2012), referrals as a result of the Aboriginal Knockout Health Challenge increased from 0.5 to 31.3%; referrals from Aboriginal health professionals decreased from 15.4 to 3.5% pre to post Aboriginal program implementation; and referrals from other health professionals increased from 8.4 to 19.9% after the implementation of the Aboriginal program.

#### Risk factor profile of GHS coaching participants

Aboriginal participants were significantly more likely to be overweight or obese compared to non-Aboriginal participants (96.3% compared to 89.5%; *p*-value <0.0001), have a waist circumference measurement that placed them at an increased or greatly increased risk of chronic disease (96.3% compared to 91.0%; *p*-value <0.0001), consume less than the recommended daily serves of fruit (61.1% compared to 52.1%; *p*-value <0.0001), not undertake the recommended levels of physical activity (62.0% compared to 65.6%; *p*-value =0.03) compared to non-Aboriginal participants (Table 2).

**Table 2:** Risk factor profile of Aboriginal and non-Aboriginal GHS coaching participants at baseline, February 2009-December 2015

	Aboriginal		Non-Aboriginal		All	
	n	%	n	%	N	%
Body Mass Index [BMI]						
Under or acceptable weight	32	3.8	2016	10.5	2048	10.2
Overweight	125	14.7	5115	26.7	5240	26.2
Obese	694	81.6	12042	62.8	12736	63.6
Waist Circumference						
No risk	26	3.7	1358	9.0	1384	8.7
Increased risk	61	8.6	2280	15.0	2341	14.7
Greatly increased risk	622	87.7	11530	76.0	12152	76.5
Fruit and Vegetable consumption						
Less than 2 serves of fruit per day	499	61.1	9977	52.1	10476	52.5
Two or more serves of fruit per day	318	38.9	9168	47.9	9486	47.5
Less than 5 serves of vegetables per day	732	89.6	16886	88.2	17618	88.2
5 or more serves of vegetables per day	85	10.4	2265	11.8	2350	11.8
Physical activity						
Insufficient physical activity	500	62.0	12298	65.6	12798	65.4
Sufficient physical activity	307	38.0	6455	34.4	6762	34.6

Waist circumference risk was computed differently for males and females. For males: increased risk ≥94 cm and <102 cm, greatly increased risk ≥102 cm; for females: increased risk ≥80 cm and <88 cm, greatly increased risk ≥88 cm [31]. Insufficient physical activity is defined as not engaging in ≥5 sessions per week of walking, or ≥5 sessions per week of moderate activity, or 3–4 sessions per week of walking and ≥1–2 sessions per week of moderate activity, or ≥1–2 sessions per week of walking and 3–4 sessions per week of moderate activity [32]

#### Outcomes of the coaching program

Overall, GHS participants who completed the 6-month coaching program made significant improvements (mean (SD)) with an average weight loss of 3.6 kg (5.0); an average decrease in waist circumference of 4.8 cm (6.6); an average decrease in BMI of 1.3 units (1.9); with increases in the number of walking and moderate and physical activity sessions (1.1 (2.9) and 0.7 (2.3) sessions respectively); and increases in fruit and vegetable consumption (0.4 (1.1) and 1.1 (1.5) serves respectively) and decreases in the consumption of sweetened drinks and takeaway meals (−0.2 (1.0) and −0.4 (1.2) serves respectively) (all *p*-value <0.0001).

Aboriginal participants also made significant improvements to their lifestyle risk factors at both three and six months (Table 3). At three months Aboriginal participants had an average weight loss of 2.1 kg; an average decrease in waist circumference of 3.4 cm; and an average decrease in BMI of 0.8 units. At six months Aboriginal participants also made significant improvements with an average weight loss of 3.3 kg; an average decrease in waist circumference of 6.2 cm; and an average decrease in BMI of 1.2 units.

**Table 3:** Anthropometric and behavioural risk factor changes from baseline to 3-months and 6-months for Aboriginal GHS coaching participants, February 2009 – December 2015

	Changes at 3 months					Changes at 6 months				
	N	Baseline [SD]	3-months [SD]	Change [SD]	*p*-value	N	Baseline [SD]	6-months [SD]	Change [SD]	*p*-value
Weight [kg]^a^	199	98.5 [23.0]	96.5 [22.6]	−2.1 [6.3]	<0.0001	103	97.3 [20.3]	94.1 [21.1]	−3.3 [9.4]	0.001
BMI [kg/m^2^]^a^	195	35.5 [7.2]	34.7 [7.2]	−0.8 [2.4]	<0.0001	101	35.7 [6.7]	34.4 [7.0]	−1.2 [3.7]	0.001
Waist circumference [cm]^a^	145	112.5 [17.2]	109.1 [18.0]	−3.4 [5.8]	<0.0001	74	111.3 [15.2]	105.1 [16.7]	−6.2 [7.8]	<0.0001
Fruit [daily serves]^b^	203	1.3 [1.1]	1.8 [1.0]	0.5 [1.0]	<0.0001	108	1.3 [1.1]	1.8 [1.0]	0.5 [1.0]	<0.0001
Vegetables [daily serves]^b^	210	2.5 [1.7]	3.2 [1.6]	0.8 [1.4]	<0.0001	108	2.6 [1.6]	3.5 [1.5]	0.9 [1.3]	<0.0001
Sweetened drinks [daily serves]^b^	202	0.6 [1.4]	0.3 [1.0]	−0.3 [1.4]	0.002	106	0.5 [1.1]	0.2 [0.9]	−0.2 [1.4]	NS
Takeaway meals [weekly serves]^b^	191	1.0 [1.4]	0.6 [1.0]	−0.3 [1.2]	<0.0001	105	0.8 [1.3]	0.5 [1.0]	−0.3 [1.0]	0.001
Walking [no. 30 min sessions per week]^b^	206	2.3 [2.6]	3.0 [2.5]	0.7 [2.7]	<0.0001	111	2.5 [2.8]	3.0 [2.7]	0.5 [3.3]	NS
Moderate Physical activity [no. 30 min sessions per week]^b^	204	0.9 [1.6]	1.5 [2.1]	0.5 [2.1]	0.001	108	1.1 [1.7]	1.8 [2.3]	0.8 [2.3]	0.001
Vigorous physical activity [no. of 20 min sessions per week]^b^	194	0.5 [1.3]	0.9 [1.7]	0.3 [1.6]	0.003	104	0.4 [1.1]	0.8 [1.6]	0.4 [1.6]	0.007

*NS* Not significant; matched pair analysis; ^a^T-test undertaken for matched paired samples for significant mean difference; ^b^Non parametric test undertaken for related samples for significant median difference

**Fig. 2:**
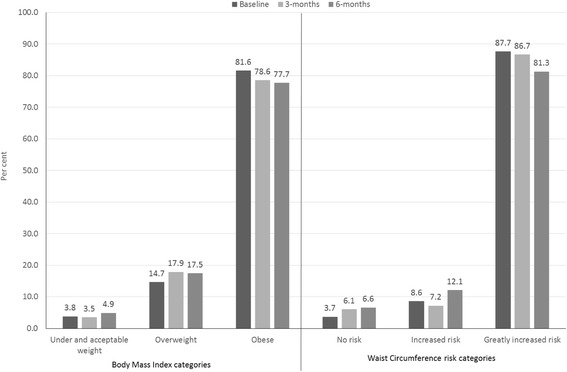
Proportion of Aboriginal participants classified as overweight and obese at baseline; and at risk due to waist circumference measurements, 3-months and 6-months, February 2009-December 2015

Aboriginal participants who completed the 6-month coaching program also made improvements to their chronic disease risk profile, with 60.8% (*n* = 62) of participants losing more than 2.5% of their initial baseline body weight. There were significant improvements from baseline to three and six months in the proportion of Aboriginal participants classified as being obese and overweight and similar improvements in the proportion of participants at risk of chronic disease due to their waist circumference (Fig. 2).

There were no significant differences between the anthropometric and behavioural risk factor improvements [between baseline and 6-months] by Aboriginal and non-Aboriginal participants who had completed the coaching program. Further, the results suggest that there were no significant differences between the anthropometric and behavioural risk factor improvements achieved by Aboriginal participants based on the program they completed [i.e. the Aboriginal specific coaching program compared to the standard GHS program] (Table 4).

**Table 4:** Change in outcomes between Aboriginal and non-Aboriginal coaching participants and Aboriginal participants’ pre-Aboriginal and post-Aboriginal program implementation, February 2009-December 2015

	Aboriginal^a^	Non-Aboriginal^a^	Pre Aboriginal program implementation^b^	Post Aboriginal program implementation^b^
	Mean [SD]	Mean [SD]	Mean [SD]	Mean [SD]
Change in weight [kg]	−3.9 [7.2]	−3.6 [4.9]	−4.7 [6.1]	−2.7 [8.6]
Change in BMI [kg/m^2^]	−1.5 [2.7]	−1.3 [1.8]	−1.7 [2.3]	−1.1 [3.1]
Change in waist circumference [cm]	−6.2 [7.8]	−4.8 [6.5]	−6.9 [7.6]	−5.0 [8.1]
Change in fruit consumption [daily serves]	0.5 [1.0]	0.3 [1.1]	0.7 [0.9]	0.3 [1.1]
Change in vegetable consumption [daily serves]	0.9 [1.4]	1.1 [1.5]	1.2 [1.4]	0.5 [1.3]
Change in takeaway consumption [weekly serves]	−0.3 [1.0]	−0.4 [1.2]	−0.5 [1.0]	−0.1 [0.9]
Change in sweetened drinks consumption [daily serves]	−0.3 [1.4]	−0.3 [0.9]	−0.3 [1.7]	−0.1 [0.7]
Change in walking [30 min sessions per week]	0.5 [3.3]	1.1 [2.9]	0.9 [2.9]	−0.2 [3.6]
Change in moderate physical activity [30 min sessions per week]	0.8 [2.3]	0.7 [2.3]	0.8 [2.0]	0.7 [2.7]
Change in vigorous physical activity 20 min [sessions per week]	0.4 [1.6]	0.4 [1.5]	0.7 [1.6]	0.1 [1.6]

Change in the variable between baseline and 6-months; an independent samples T-test of significance was undertaken comparing participant’s change between baseline and follow up; no results of significance were found

^a^ *n* = range of 108–74 for Aboriginal participants; *n* = range of 5,455-5,272 for Non-Aboriginal participants

^b^ *n* = range of 66–60 for Aboriginal participants pre-program implementation; *n* = range of 45–39 for Aboriginal participants post-program implementation

## Discussion

This study demonstrates that a community consultation process in partnership with Aboriginal communities can lead to the enhancement of a mainstream service which meets the health and cultural needs of the population. It also demonstrates that strategies to ensure culturally accessible services are available for Aboriginal people should: involve the community in planning and delivering services, respect and respond to differences in culture; incorporate flexibility and be well-coordinated; train non-Aboriginal staff in cultural competency skills and engage Aboriginal specific health services for delivery and promotion [17–20, 36, 38, 39].

The formative research and subsequent service delivery enhancements to promote the GHS Aboriginal program were effective at increasing the reach of the service. The initiatives undertaken to build awareness and credibility of the enhanced GHS for Aboriginal people through community promotions and networks [37] as well as partnerships with the ACCHS to promote the service locally, were effective and tripled the participation rate of Aboriginal people in the GHS. ACCHS have been identified through social network analysis as having a central role in communicating health information through Aboriginal communities [37]. However, clearly finding a way to improve referral rates from Aboriginal health workers in particular, including strategies to educate and train these staff in the use of the GHS [40], might help sustain or further improve recruitment of Aboriginal people into the GHS.

The mainstream GHS has historically attracted recruitment and participation from adult females in comparison to males [16], but this is a commonly recognised demographic who readily participate in health coaching programs nationally and internationally [16]. However, given the reported disparity in the prevalence of certain chronic diseases even between Aboriginal females and males e.g. rates of diabetes [3], continued support to help Aboriginal females to complete the GHS will potentially help contribute to a further population level reduction in chronic diseases. Further formative research into the most effective strategies to recruit and retain Aboriginal males into the GHS is needed.

Interestingly, the Aboriginal participants enrolling and completing the enhanced GHS are demographically different to the Aboriginal participants completing the mainstream program. Aboriginal participants registered in the enhanced GHS versus the mainstream program were significantly more likely to be classified in the lowest SEIFA quintiles (85.9% compared to 66.1%; *p*-value <0.0001) and be from regional, remote and very remote locations (63.1% compared to 40.2%; *p*-value <0.0001). These findings suggest that the enhanced service is targeting and reaching the most disadvantaged members of Aboriginal communities and may help increase access to weight loss coaching programs for people with limited resources and who might be otherwise be geographically or socially isolated.

The impact of the GHS reported in this study (both the standard GHS program and the Aboriginal specific program) on the anthropometric and behavioural risk factors of its Aboriginal participants have been positive and in line with previous research [15]. The results also demonstrate that Aboriginal participants experience the same magnitude of improvement to their lifestyle risk factors as non-Aboriginal participants.

Aboriginal participants who completed the Aboriginal specific program [when it was introduced] did not demonstrate any greater improvements than Aboriginal participants who completed the standard GHS program. However, the utility of the enhanced Aboriginal GHS cannot be measured purely on quantitative health outcomes, as it is the design, delivery and promotion of the Aboriginal specific program that has led to greater acceptability and accessibility of the program in the Aboriginal community. However, further strategies to increase service efficiency and effectiveness will be needed over time to help reduce (not widen) the equity gap in the behavioural risk factors for chronic disease between Aboriginal and non-Aboriginal people. These might include employing GHS coaches who identify as being of Aboriginal and/or Torres Strait Islander descent and promotion of the enhanced GHS through local Aboriginal men’s and women’s groups.

In relation to the quantitative study, a number of limitations are noteworthy, including the use of self-report data. Anthropometric and behavioural outcome data relies on self-report which is likely to include social desirability biases and general inaccuracies. Despite a number of quality checks in place to improve data capture, missing data is evident [possibly through incomplete questionnaires and errors in data entry] which potentially introduces some bias worth acknowledging. Further, a change in service provider in January 2014 resulted in variations in the evaluation datasets. Efforts have been made to standardise the data collection process, but the potential for bias both in the delivery of the GHS and the collection of evaluation data must again be acknowledged.

This study provides one of a few examples where a mainstream service has been adapted to effectively meet the health and cultural needs of Aboriginal communities, contributing to a reduction in risk of chronic diseases for this population in NSW. With the growing evidence base [38, 41–44] regarding effective strategies to redesign services that are culturally appropriate, the field of chronic disease prevention must continue to seek opportunities to enhance service provision to meet the needs of Aboriginal communities.
